# Reverberant magnetic resonance elastographic imaging using a single mechanical driver

**DOI:** 10.1088/1361-6560/acbbb7

**Published:** 2023-02-27

**Authors:** Irteza Enan Kabir, Diego A Caban-Rivera, Juvenal Ormachea, Kevin J Parker, Curtis L Johnson, Marvin M Doyley

**Affiliations:** 1 University of Rochester, Hajim School of Engineering and Applied Sciences 1467, Rochester, NY, United States of America; 2 University of Delaware, Department of Biomedical Engineering 19716, Newark, DE, United States of America; 3 Verasonics, Inc., 11335 NE 122nd Way, Suite 100 98034 Kirkland, WA, United States of America

**Keywords:** magnetic resonance elastography, magnetic resonance imaging, elastography, reverberant shear wave, brain imaging, phantom

## Abstract

Reverberant elastography provides fast and robust estimates of shear modulus; however, its reliance on multiple mechanical drivers hampers clinical utility. In this work, we hypothesize that for constrained organs such as the brain, reverberant elastography can produce accurate magnetic resonance elastograms with a single mechanical driver. To corroborate this hypothesis, we performed studies on healthy volunteers (*n* = 3); and a constrained calibrated brain phantom containing spherical inclusions with diameters ranging from 4–18 mm. In both studies (i.e. phantom and clinical), imaging was performed at frequencies of 50 and 70 Hz. We used the accuracy and contrast-to-noise ratio performance metrics to evaluate reverberant elastograms relative to those computed using the established subzone inversion method. Errors incurred in reverberant elastograms varied from 1.3% to 16.6% when imaging at 50 Hz and 3.1% and 16.8% when imaging at 70 Hz. In contrast, errors incurred in subzone elastograms ranged from 1.9% to 13% at 50 Hz and 3.6% to 14.9% at 70 Hz. The contrast-to-noise ratio of reverberant elastograms ranged from 63.1 to 73 dB compared to 65 to 66.2 dB for subzone elastograms. The average global brain shear modulus estimated from reverberant and subzone elastograms was 2.36 ± 0.07 kPa and 2.38 ± 0.11 kPa, respectively, when imaging at 50 Hz and 2.70 ± 0.20 kPa and 2.89 ± 0.60 kPa respectively, when imaging at 70 Hz. The results of this investigation demonstrate that reverberant elastography can produce accurate, high-quality elastograms of the brain with a single mechanical driver.

## 1. Introduction

Magnetic resonance elastography (MRE) is an imaging technique for determining the mechanical properties of tissues noninvasively and *in vivo* (Muthupillai *et al* 1995), and the current gold standard imaging method for diagnosing liver fibrosis (Yin *et al* 2007, Venkatesh *et al* 2013). Given the success of MRE in this application, several studies are now focused on investigating MRE’s utility in other clinical applications. These include improving the differential diagnosis of breast cancer (McKnight *et al* 2002, Sinkus *et al* 2007, Patel *et al* 2021), identifying tears in skeletal muscles, (Dresner *et al* 2001), detecting pulmonary disease in lungs (Mariappan *et al* 2014), and diagnosing prostate cancer (Brock *et al* 2015). MRE could also prove helpful in assessing the progression of Alzheimer’s disease (Murphy *et al* 2011), Parkinson’s disease (Lipp *et al* 2013), multiple sclerosis (Wuerfel *et al* 2010), brain integrity and microstructural changes in health and disease (Sack *et al* 2013), and evaluating normal pressure hydrocephalus (Streitberger *et al* 2011, Freimann *et al* 2012). Li and colleagues reviewed the crucial elements common to all successful magnetic resonance elastographic imaging systems (Li *et al* 2014): appropriate mechanical stimulation of the organ under investigation, acquiring wave images with a good signal-to-noise ratio (SNR), and computing robust estimates of shear modulus from the measured wave fields. In this work, we focus on the third element, i.e. efficiently computing shear modulus.

Researchers have proposed different approaches for computing shear modulus that vary in accuracy and computational efficiency (Doyley 2012). One method computes shear modulus directly from local estimates of wavelengths (Manduca *et al* 1996). Although this is computationally efficient, estimating the wavelength in complex organs, such as the brain, is difficult because waves reflecting from the skull and internal structures superimpose to create complex shear wave fields (Muthupillai *et al* 1995). The local frequency estimator (LFE) is an alternative method for estimating shear modulus (Knutsson *et al* 1994, Kruse *et al* 2000, Hu 2020). Like the local wavelength estimation (LWE) approach, the LFE method is computationally efficient but produces erroneous shear modulus when applied to complex wave fields (Hiscox *et al* 2016). Hu *et al (* 2020 *)* proposed an enhanced local frequency estimator (ELFE) that used directional filters to eliminate undesirable reflections. They demonstrated that ELFE produced more accurate shear modulus estimates than the conventional LFE approach and reduced far-field artifacts (i.e. artifacts in regions far from the wave source). Researchers have proposed several direct inversion algorithms to overcome challenges incurred when estimating shear modulus with either LWE or the LFE approach by algebraically solving for the complex shear modulus from the Helmholtz equation (Manduca *et al* 2001, Oliphant *et al* 2001, Papazoglou *et al* 2008, Barnhill *et al* 2018). Although these direct inversion schemes are fast and accurate, they are more susceptible to noise than the LFE, LWE, or ELFE methods (Hu 2020) investigated the impact of measurement range on two shear modulus estimation approaches, the algebraic-inversion-of-differential-equation (AIDE) and the local frequency estimator. Using the wavelength-to-pixel size ratio performance metric, they revealed that AIDE incurred significant errors when the wavelength-to-pixel ratio was less than 10. In contrast, the LFE method incurred errors only when the wavelength-to-pixel ratio was less than 2, showing its superiority over the AIDE method. To improve the robustness of the direct inversion method (Barnhill *et al* 2018), developed a multi-frequency inversion approach that incorporates first-order gradients and combines shear modulus estimates from a narrow range of frequencies. Researchers have also used filtering schemes to enhance performance (Scott *et al* 2020). However, excessive filtering degrades spatial resolution. An artificial neural network has recently been used to reconstruct the shear modulus distribution (Scott *et al* 2020). Neural networks should provide fast and reliable shear modulus estimates once the neural network is sufficiently trained; however, their performance in different clinical scenarios has yet to be revealed. Iterative inversion methods offer the opportunity to model heterogeneous, viscoelastic tissues appropriately (Van Houten *et al* 1999, 2001a, Doyley *et al* 2000, 2010). This inversion approach is robust but computationally expensive, requiring several hours to compute high-resolution elastograms. Our long-term objective is to integrate MRE into our clinical workflow. More specifically, to develop methods to provide accurate MR shear modulus elastograms at the MR console when imaging the brain.

This paper revisits the local wavelength estimation approach by considering the shear modulus estimation problem as a reverberant problem. More specifically, we seek to estimate the local wavelength of complex wave fields using a technique known as reverberant elastography. Reverberant elastography uses multiple point sources to generate complex wave fields (Parker *et al* 2017); the resulting wave fields’ autocorrelation function provides reliable local wavelength estimates. However, utilizing many mechanical drivers to produce complex wave fields can hamper clinical utility. More specifically, performing MRE with multiple drivers is impractical for many clinical applications and could prove uncomfortable for patients. In this work, we hypothesize that we can produce reliable elastograms with a single mechanical driver in constrained organs such as the brain, where complex wave fields are generated naturally. This hypothesis was based on the observation that the skull has many surfaces that act as point mechanical sources (Clayton *et al* 2012, Smith *et al* 2020). To corroborate this hypothesis, we performed studies on a constrained gelatin phantom and healthy volunteers (*n* = 3). We used a similarity metric to quantify the degree of reverberance induced in different displacement components. We evaluated the performance (accuracy) of reverberant elastograms relative to those computed using the subzone inversion method (Van Houten *et al* 2001a, Doyley *et al* 2010). Also, we assessed the feasibility of recovering shear modulus from a single component of the reverberant wave field, because doing so would overcome a limitation of the subzone inversion method that requires the entire 3D displacement field to produce reliable elastograms. Currently, the 3D displacement field is acquired by applying a phase contrast pulse sequence (Weaver *et al* 2001) three times, one for each component. Reverberant elastography could reduce MRE acquisition time (by a factor of three) if it produced accurate elastograms from a single displacement component.

## 2. Materials and methods

### 2.1. Reverberant shear wave elastography

The general principles of reverberant elastography have been previously described (Ormachea *et al* 2018, 2019, Zvietcovich *et al* 2019, Ormachea and Parker 2021); therefore, in this section, we provide a summary of the approach. Complex wave fields are produced when plane waves that originate from multiple point sources or reflected from various angles superimpose. The particle velocity *V* (*ε*, t) of the complex wave field is given by Parker *et al* (2017):

\begin{eqnarray*}V\left(\varepsilon ,\,t\right)=\displaystyle \sum _{q,l}{\hat{n}}_{ql}{v}_{ql}{e}^{i\left({k}_{1}.\varepsilon -{\omega }_{0}t\right)},\end{eqnarray*}

where *t* and *ε* represent the time and position in the complex (reverberant) wave field, respectively; *k* represents the wavenumber, and *ω*_0_ the angular frequency. The subscript *q* denotes the random unit vector, and the subscript $l$ denotes a unit vector, ${n}_{ql},$ parallel to a disk formed by orthogonal basis vectors $\hat{\varnothing }$ and $\hat{\phi }$ (Parker *et al* 2017). The random variable ${v}_{ql}$ describes the particle velocity magnitude. For isotropic mediums, the autocorrelation of the wave field in a plane transverse to the detected motion vector is given by (Parker *et al* 2017):

\begin{eqnarray*}{B}_{\mathrm{vv}}\left({\mathrm{\Delta }}{\epsilon }_{x}\right)=\frac{\beta }{2}\left[{j}_{0}\left(k{\mathrm{\Delta }}{\varepsilon }_{x}\right)-\frac{{j}_{1}\left(k{\mathrm{\Delta }}{\varepsilon }_{x}\right)}{\left(k{\mathrm{\Delta }}{\varepsilon }_{x}\right)}\right],\end{eqnarray*}

where *B*_*vv*_ represents the 2D autocorrelation of *V* (*ε*, t), ${\mathrm{\beta }}$ represents the expected value of the squared particle velocity magnitude, *k* is the wavenumber, and j_0_ and j_1_ are spherical Bessel functions of the first kind of order 0 and 1, respectively. Since ${n}_{ql}$ and ${v}_{ql}$ are independent of position, an ersatz form of equation (1) can be written as:

\begin{eqnarray*}{\mathrm{\nu }}\left(\epsilon \right)={{\mathrm{V}}}_{{\mathrm{o}}}{{\mathrm{e}}}^{{\mathrm{i}}\varnothing \left(\epsilon \right)},\end{eqnarray*}

where $\varnothing \left(\epsilon \right)$ represents the spatially varying phase, and *V*_o_ is related to the root mean squared amplitude of the field. The wavenumber, *k*, is computed from the ensemble average of the reverberant field as follows:

\begin{eqnarray*}{k}^{2}={\mathrm{A}}{\left|\displaystyle \frac{{\mathrm{d}}\varnothing }{{\mathrm{d}}{\mathrm{\varepsilon }}}\right|}^{2},\end{eqnarray*}

where *A* is a scaling constant, which was determined empirically to be one in this study using known measurement of shear wave speed. The bracket denotes the average value over a homogeneous kernel.

In this study, we acquired MR motion data over four-time points or equally spaced phase offsets. The Fourier transform of these temporal data provided complex motion at the vibration frequency. We computed the phase angle of the motion data at each pixel in the imaging field of view (FOV). Assuming phase varies in three dimensions:

\begin{eqnarray*}\varnothing \left(x,y,z\right)\cong {k}_{x}x+{k}_{y}z+{k}_{z}z+{c}_{0},\end{eqnarray*}

where *k_x_*, *k_y_* and *k_z_* are components of the wave vectors in the *x*, *y* and *z* coordinate directions, respectively, and *c*_o_ is a constant. Substituting equation (5) into equation (4) gives an approximate estimate of the wavenumber:

\begin{eqnarray*}{k}^{2}=A\left({{k}_{x}}^{2}+{{k}_{y}}^{2}+{{k}_{z}}^{2}\right).\end{eqnarray*}

We applied the two-dimensional unwrapping algorithm described in Zhao *et al *(2018) to phase maps acquired from each coordinate direction. We computed local estimates of wave number by using the singular value decomposition method (Strang 2016) to fit a plane to the unwrapped phase maps within three-dimensional overlapping kernels. Shear wave speed (c) was computed from local estimates of wavenumber as follows:

\begin{eqnarray*}c=\displaystyle \frac{2\pi f}{k},\end{eqnarray*}

where *f* represents the shear wave frequency. Local estimates of shear modulus, $\mu $ *,* were computed from the estimated shear wave speed as follows (Parker *et al* 2010, 2011):

\begin{eqnarray*}\mu =\rho {c}^{2},\end{eqnarray*}

where, ρ represents tissue density (1 g cm^−3^). In this study, shear wave speed was assumed to be isotropic. The shear modulus of the tissue or phantom under investigation was estimated by applying the reverberant method to each phase direction and then computing the average of the resulting images (i.e. the composite shear modulus elastograms).

### 2.2. Subzone elastography

The overlapping subzone inversion method computes shear modulus elastograms from MR-measured internal tissue displacements by combining the finite element method and the Newton-Raphson iterative scheme, previously described (Van Houten *et al* 1999, 2001b, Doyley *et al* 2003). This inversion approach seeks the distribution of mechanical parameters (in our case, lambda (*λ*) and shear (*μ*) modulus) that minimize the difference between internal tissue displacements calculated with the finite element model and those measured with MR. To reduce the memory required to solve the three-dimensional inverse elasticity problem on high-resolution finite element meshes, the solution domain was divided into a series of computationally independent overlapping subdomains, as described in Van Houten *et al *(2001b). The objective function that was minimized at the subzone level is given by

\begin{eqnarray*}\varphi \left({\mu }_{{\mathrm{z}}},{\lambda }_{{\mathrm{z}}}\right)=\parallel {{\bf{U}}}^{z}\left({{\boldsymbol{\mu }}}_{z},{{\boldsymbol{\lambda }}}_{z}\right)-{{\bf{U}}}_{m}^{z2}\parallel ,\end{eqnarray*}

where **U** ^z^ (***μ***_*z*_, ***λ***_*z*_) and **U** ^z^_*m*_ represent vectors of the calculated and measured displacements at the nodal coordinates of each subzone, respectively. ***μ***_z_ and ***λ***_z_ represent shear and lambda modulus at the nodal coordinates of each subzone, respectively. Setting the derivative of equation (9) with respect to ***μ***_z_ and ***λ***_z_ to zeros, and solving the resulting nonlinear equations with the Newton-Raphson iterative scheme, gives the resulting matrix solution at the (*t*+1) iteration:

\begin{eqnarray*}\begin{array}{l}{\{{\mu }_{z},{\lambda }_{z}\}}^{t+1}={\{{\mu }_{z},{\lambda }_{z}\}}^{t}+{\left[{J}_{z}^{T}\left({\mu }_{{\mathrm{z}}},{{\boldsymbol{\lambda }}}_{{\mathrm{z}}}\right){J}_{z}\left({\mu }_{{\mathrm{z}}},{{\boldsymbol{\lambda }}}_{{\mathrm{z}}}\right)+{\boldsymbol{I}}{\mathrm{\alpha }}\right]}^{-1}\\ \left.\cdot \,{J}_{{\mathrm{z}}}^{{\mathrm{T}}}\left({U}_{{\mathrm{m}}}^{{\mathrm{z}}}-U\left({\mu }_{{\mathrm{z}}},{{\boldsymbol{\lambda }}}_{{\mathrm{z}}}\right)\right)-{\mathrm{\alpha }}{\{{\mu }_{z},{\lambda }_{z}\}}^{t}\right],\end{array}\end{eqnarray*}

where **J**_z_ (***μ***_z_, ***λ***_z_) is the *n* × *n* Jacobian matrix, and $\alpha $ is a positive number that was used to improve the condition of the Hessian matrix, [**J** ^T^_z_ (***μ***_z_, ***λ***_z_) **J**_z_ (***μ***_z_, ***λ***_z_)].

### 2.3. Phantom experiments

This study aimed to show that reverberant elastography produces reliable shear modulus estimates from complex wave fields induced in constrained objects, with multiple reflecting surfaces using a single mechanical driver. To demonstrate this, we compared the performance (accuracy and contrast-to-noise ratio (CNR)) of reverberant elastograms to subzone elastograms. We also explore the feasibility of recovering shear modulus from a single component of the reverberant wave field. Independent mechanical testing provided absolute shear modulus values.

#### 2.3.1. Phantom fabrication

**Figure 1.**
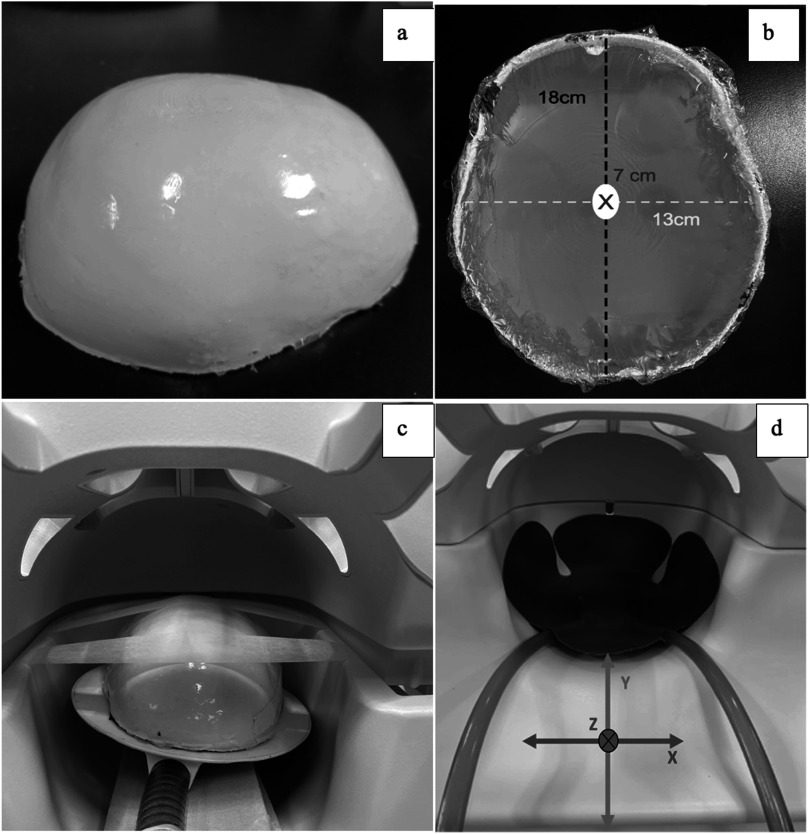
(a) Photograph showing the elastic brain-shaped phantom used in the experimental studies (side view), (b) Top view of the phantom shown in (a). (c) Experimental setup used for phantom imaging, showing the 20-channel head coil used for MR imaging and the pneumatic mechanical driver used to induce shear waves within the phantom. The pillow driver (blue insert) used for clinical imaging is shown in (d).

We fabricated a brain-shaped phantom (180 mm (long axis) × 130 mm (short axis) × 70 mm (height), see figures 1(a), (b)) from a suspension consisting of bovine gelatin (200 bloom; Sigma Aldrich Chemicals, St. Louis, MO, USA), de-ionized water (18 MΩ), and ethylenediamine tetra-acetic acid (Sigma Aldrich Chemicals, St. Louis, MO, USA) in a highly controlled and repeatable manner as described in Doyley *et al* (2003). The phantom contained three spherical gelatin inclusions with diameters of 18 mm, 12 mm and 4 mm. Table 1 gives the percentage by weight of the gelatin, water, and copper sulfate used to fabricate the surrounding background and inclusions.

**Table 1.** Concentration by weight of the materials used to fabricate elasticity phantom.

	Gelatin (%)	Water (%)	Copper-sulphate (%)	Actual shear modulus (kPa)
Background	8	92	0	3.34 ± 0.04
Inclusions	18	81.64	0.36	8.15 ± 0.05

#### 2.3.2. Elastographic imaging

We performed all elastographic imaging in a whole body 3T MRI scanner (Prisma, Siemens, Erlangen, Germany) with a 20-channel head coil. A pneumatic actuator with a passive driver (Resoundant, Inc., Rochester, MN, USA) was used to induce shear waves in each phantom, as illustrated in figure 1(a). We used two vibration frequencies (50 Hz and 70 Hz) separately during elastographic imaging, with motion-encoding gradients matched in the period to vibration and with a variable number of gradients depending on frequency. It took approximately six minutes to acquire each 3D data set. Table 2 summarizes the actuator amplitude, gradient amplitude, and the number of gradients used at each frequency. The single-shot echo-planar imaging (EPI) sequence (Johnson *et al* 2014, Chaze *et al* 2019) measured the resulting time-varying harmonic tissue displacements. We configured the MR scanner with echo and repetition times of 76.0 ms and 8640 ms, respectively. Forty axial slices were acquired for the phantom with a 153 mm × 153 mm × 60 mm field-of-view (1.6 mm isotropic voxel size), with four images with relative phase offsets. For brain, eighty axial slices with four phase offsets were acquired with a 240 mm × 240 mm × 120 mm field-of-view (2.5 mm isotropic voxel size). We used the Fourier transform method described in Sinkus *et al *(2000) to estimate the complex, three-dimensional displacement field. To remove low-frequency longitudinal waves and high-frequency noise, we applied a two-dimensional bandpass filter in all directions. The cutoff spatial frequency related to the wavenumber k of the filter was determined from pre-selected low (*c*_l_) and high (*c*_h_) shear wave speed values. The corresponding filter cutoffs were *k*_l_ = (2 × *k* × *π* × *f*)/*c*_h_, and *k*_h_ = (2 × *π* × *f*)/*c*_l_. For this study *c*_h_ was 3.5 m s^−1^, and *c*_l_ was 0.3 m s^−1^. These values were selected using phantoms with known shear modulus and an average shear modulus of the whole brain reported in the literature.

**Table 2.** MRE scanning parameters used in the phantom studies.

Frequency (Hz)	Actuator amplitude (%)	Gradient amplitude (mT/m)	Number of gradients
50	6	8	1
70	14	20	2

Two groups of modulus elastograms were computed from each data set; one was computed using the reverberant shear modulus estimation method and the other with the subzone reconstruction method. The reverberant method was performed using 6.4 mm × 6.4 mm × 6.4 mm overlapping kernels. All subzone reconstructions were conducted on a finite element mesh consisting of 70 000 nodes and 415 000 elements (created using MATLAB version R2022b). A spatial filtering weight of 20% and subzone radius size of 9 mm and a regularization value of 1 e^−7^ were also employed during subzone reconstructions. A homogeneous trial solution (shear and lambda moduli of 0.33 kPa and 33 kPa, respectively) was assumed at the start of all subzone reconstructions. Reconstructions were terminated either after 100 global iterations or when the relative error of the global objective function did not decrease significantly (2%) for 10 consecutive iterations, whichever condition occurred first. In general, it took 7 h to compute subzone elastograms and 3 min to compute reverberant elastograms on an Intel Xeon Gold 6330 CPU computer system (20 cores) running at 2 GHz (Dell Technologies, Round Rock, Texas USA).

#### 2.3.3. Mechanical testing

We used a Landmark Servo Hydraulic Test System (MTS, Eden Prairie, MN, USA) for the independent mechanical tests. We made cylindrical samples (19 mm diameter × 10 mm height) from the same batch of gelatinous suspension used to manufacture the inclusions and surrounding tissue. Each cylindrical sample was deformed by applying stresses ranging from 0 to 1 kPa and Young’s modulus from the resulting stress-strain curve. Shear modulus $\mu $ was calculated from Young’s modulus (E) as follows (Fung 1981):

\begin{eqnarray*}\mu =\displaystyle \frac{E}{2\left(1+v\right)},\end{eqnarray*}

where $v\,$is the poison’s ratio, assumed to be 0.495 in this work. The ground truth for the quantitative evaluations was computed from the average of five statistically independent measurements. The actual shear modulus was 8.15 ± 0.05 kPa for the inclusions and 3.34 ± 0.04 kPa for the surrounding gel, which was consistent with previously reported values for brain tissues (Kruse *et al* 2008, Clayton *et al* 2012, Johnson *et al* 2014).

#### 2.3.4. Performance metrics

Elastograms from each reconstruction method were visually inspected for quality, and four quantitative metrics were used to evaluate performance. The octahedral shear strain signal-to-noise ratio (OSS-SNR) (McGarry *et al* 2011) was used to evaluate the quality of the measured displacement fields. The quality of the modulus elastograms was evaluated quantitatively by computing the contrast-to-noise ratio (CNR) performance metric. CNR was defined on a logarithmic scale as follows (Techavipoo and Varghese 2005):

\begin{eqnarray*}CNR\left(dB\right)=20\,\mathrm{log}\left(2\displaystyle \frac{{\left({\mu }_{b}-{\mu }_{i}\right)}^{2}}{{\sigma }_{b}^{2}+{\sigma }_{i}^{2}}\right),\end{eqnarray*}

where *μ*_b_ and *μ*_i_ represent the mean shear modulus chosen from regions-of-interest (ROIs) in the background and inclusion, respectively, while *σ*_b_ and *σ*_i_ represent the standard deviation of the shear modulus in the corresponding ROIs. The mean error (ME) performance metric was used to evaluate the accuracy of the recovered modulus compared to mechanical testing as follows:

\begin{eqnarray*}ME\left( \% \right)=\left(\displaystyle \frac{{\mu }_{{\mathrm{e}}}-{\mu }_{ref}}{{\mu }_{ref}}\right)\times 100,\end{eqnarray*}

where *μ*_e_ and *μ*_ref_ represent the estimated and from mechanical testing shear modulus, respectively.

The similarity (χ) between the measured and theoretically derived autocorrelation (**see** equation (2)) was used to quantify each displacement component’s reverberance level. For a given window, the similarity metric was computed as follows

\begin{eqnarray*}\chi \left( \% \right)=1-\sqrt{\displaystyle \frac{{\sum }_{i=1}^{N}{\left({x}_{i}-{y}_{i}\right)}^{2}}{N}}\times 100,\end{eqnarray*}

where *x_i_* and *y_i_* represent vectors of the measured and theoretically computed autocorrelation function, and *N* is the number of elements in each vector. We used a threshold to classify wave fields as complex (reverberant) or directed. This threshold was determined empirically by simulating complex wave fields created with increasing numbers of incident shear waves (i.e. from 10 to 100 with an increment of 10), as described in (Zvietcovich *et al* 2019). For each wave field, we computed the autocorrelation function of the simulated wave field and those computed theoretically using equation (2). We observed that the similarity metric increased rapidly as the number of incident shear waves increased from 10 to 50, then plateaued to 80% with a further increase in incident waves (not shown). 80% represents the threshold for differentiating fully reverberant (*n* = 100) from non-reverberant wave fields (*N* < 50). Therefore, in this study, pixels in the complex wave field whose similarity matrix (*χ*) exceeded 80% were deemed reverberant.

### 2.4. Clinical study

To evaluate the performance of reverberant elastograms under standard clinical conditions, we performed brain MRE imaging on three healthy volunteers. We acquired two datasets from each volunteer using an imaging protocol approved by the University of Delaware Institutional Review Board. In one acquisition, we induced 50 Hz shear waves within the brain. Without moving the volunteers, we increased the frequency of the induced shear waves to 70 Hz. In both acquisitions, an inflatable pillow (see figure 1(b)) positioned at the base of the skull generated shear waves within the brain. Table 3 provides the scanning parameters used in clinical imaging; all other imaging parameters are consistent with the phantom experiment. We used the FMRIB Software Library (FSL), i.e. FLIRT registration and FAST segmentation toolboxes (Smith *et al* 2004), to register and segment brain elastograms.

**Table 3.** MRE scanning parameters used in the clinical studies.

Frequency (Hz)	Actuator amplitude (%)	Gradient amplitude (mT m^−1^)	Number of gradients
50	13	70	1
70	25	70	1

## 3. Results

The proceeding subsection reports the results of experiments conducted on heterogeneous phantoms and volunteers to evaluate the performance of reverberant elastograms relative to subzone elastograms.

### 3.1. Phantom studies

#### 3.1.1. Quantitative assessment of induced displacement fields when imaging at 50 *Hz* and 70 *Hz*

**Figure 2.**
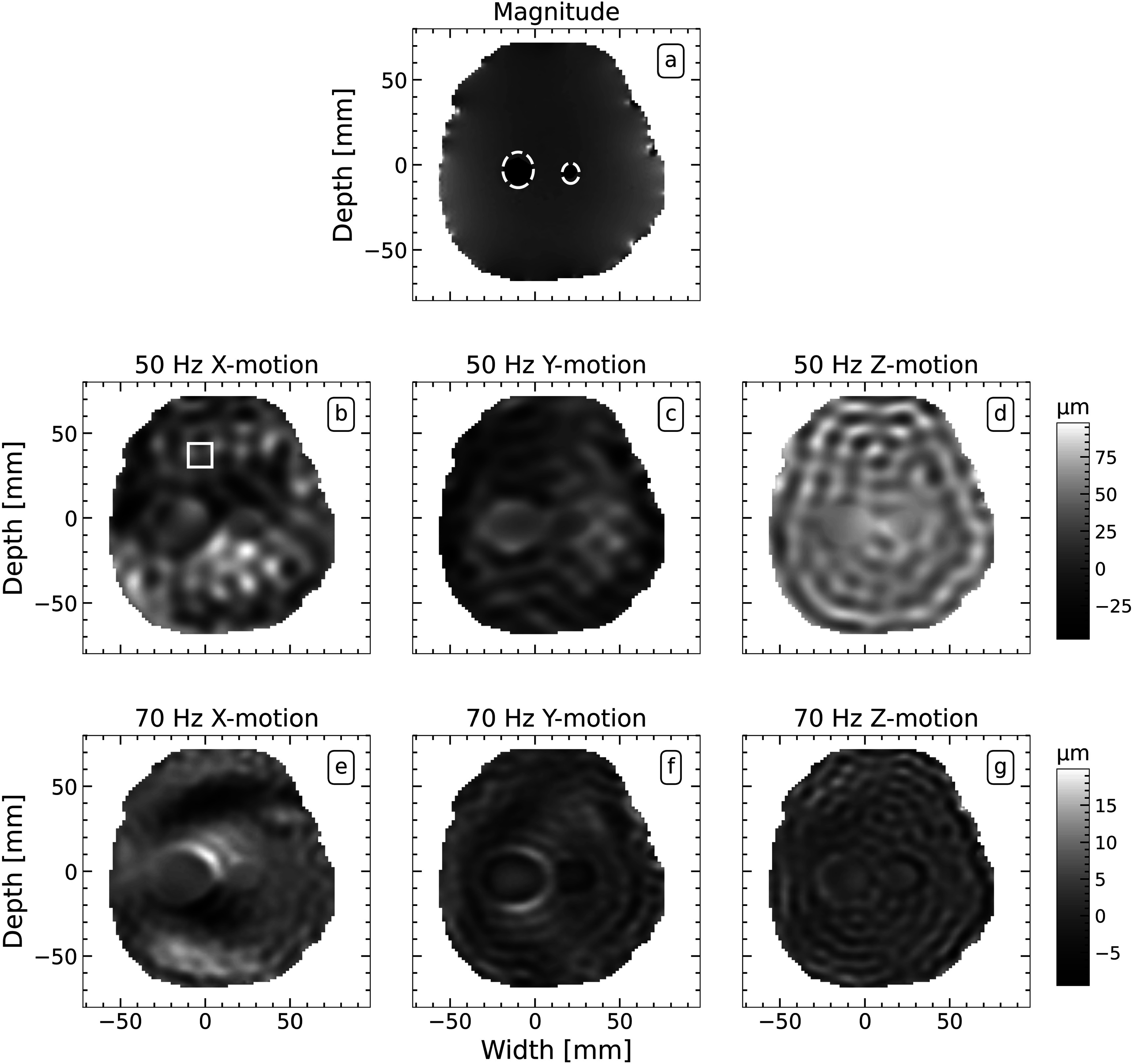
Images obtained from the central plane of a partially constrained phantom containing 18 mm, 12 mm and 4 mm diameter spherical inclusions. (a) MR magnitude image and time-harmonic displacements obtained when performing elastographic imaging with vibrations frequencies of 50 Hz (b)–(d) and 70 Hz (e)–(g).

**Figure 3.**
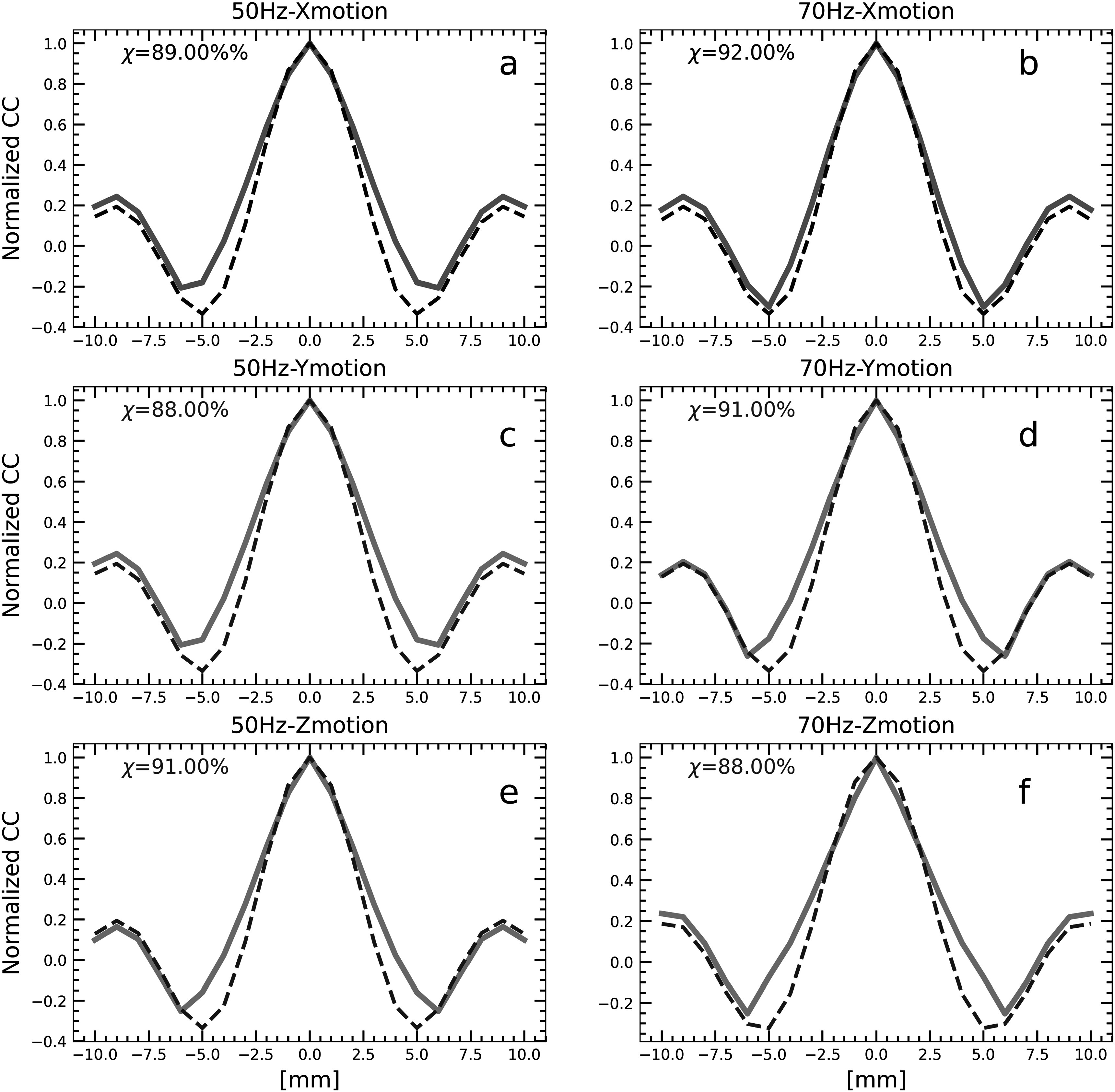
Measured and theoretically computed autocorrelation profiles within the white rectangular box region shown on figure 2(b) when imaging at 50 Hz (a), (c), (e) and 70 Hz (b), (d), (f). The solid lines represent the measured autocorrelation profiles, and the dashed line represents the theoretical profiles computed from the X (blue), Y(red), Z(green) displacement components.

Figures 2(b)–(g) shows representative displacement maps obtained from the central slice of a phantom containing spherical inclusions with diameters of 4 mm, 12 mm and 18 mm. Only two inclusions (12 mm and 18 mm diameter) were visible in the MR magnitude image (figure 2(a)). Performing elastographic imaging at 50 and 70 Hz produced time-harmonic displacements (see figures 2(b)–(g)) with OSS-SNR values of 54 and 51, respectively. Figures 3(a)–(f) shows examples of transects in the X, Y, and Z axes of the measured and theoretically derived autocorrelation functions corresponding to the rectangular region shown in (figure 2(b)) when imaging at 50 Hz ((a), (c), (e)) and 70 Hz ((b), (d), (f)). The percentage of reverberant pixels (i.e. pixels whose similarity matrix exceeded 80%) in the X, Y, and Z displacement components was 89%, 88% and 91%, respectively, when imaging at 50 Hz. Similarly, the percentage of reverberant pixels in the X, Y, and Z displacement components was 92%, 91% and 88%, respectively, when imaging 70 Hz. Although the measured wave field appears to be more reverberant when imaging at 70 Hz, statistical evaluation of the wave fields computed with the Kolmogorov-Smirnov test showed no statistically significant difference (*p* > 0.05) in wave fields obtained at 50 and 70 Hz.

#### 3.1.2. Assessment of reverberant (composite and those computed from a single displacement component) and subzone elastograms

**Figure 4.**
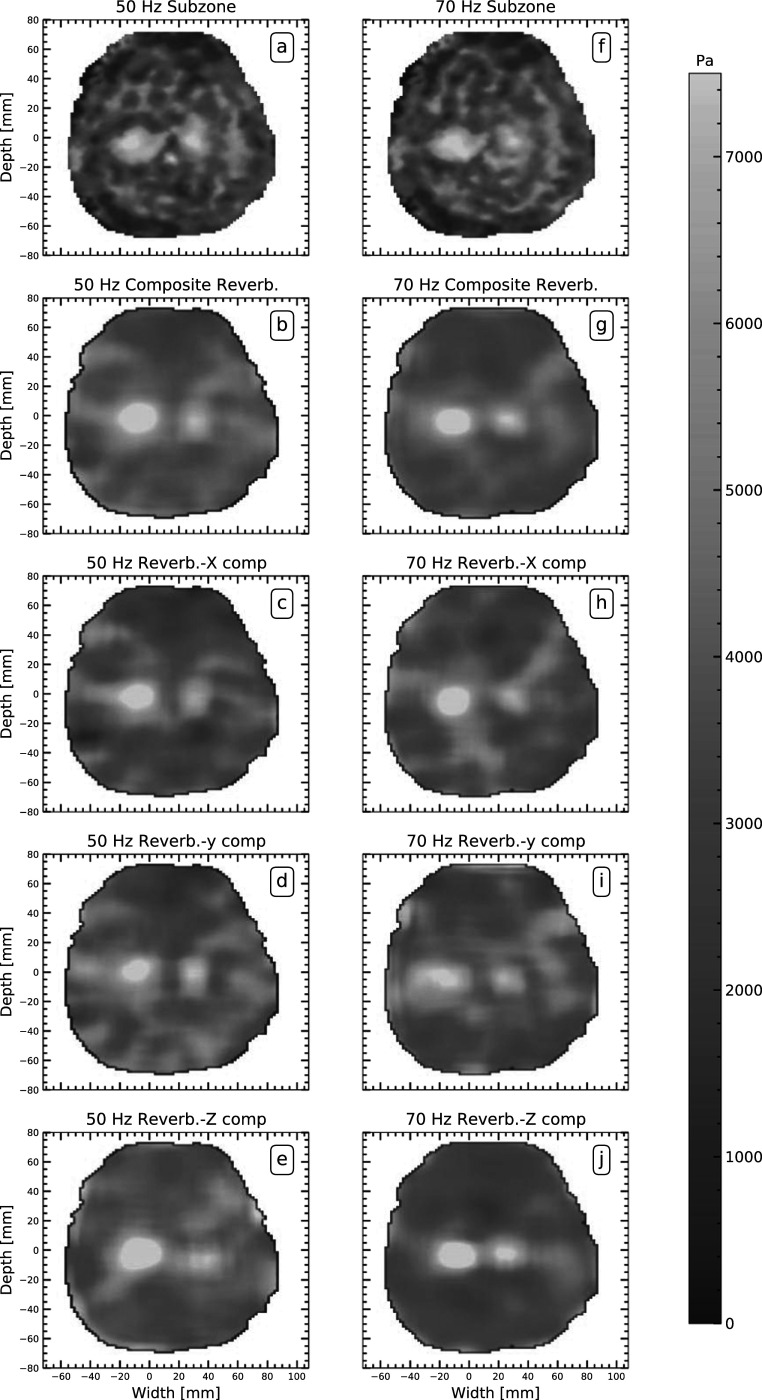
Shear modulus elastograms reconstructed from the motion fields shown in figure 3 in units of [Pa] using the reverberant method and overlapping subzone inversion method. The first row (a), (f) shows examples of elastograms computed by applying the subzone inversion method to displacements obtained when imaging at 50 Hz and 70 Hz, respectively. The second row (b), (g) shows the corresponding composite reverberant elastograms computed from individual displacement components. The remaining rows (3–5) show reverberant elastograms calculated from each displacement component (x), (y), and (z).

**Figure 5.**
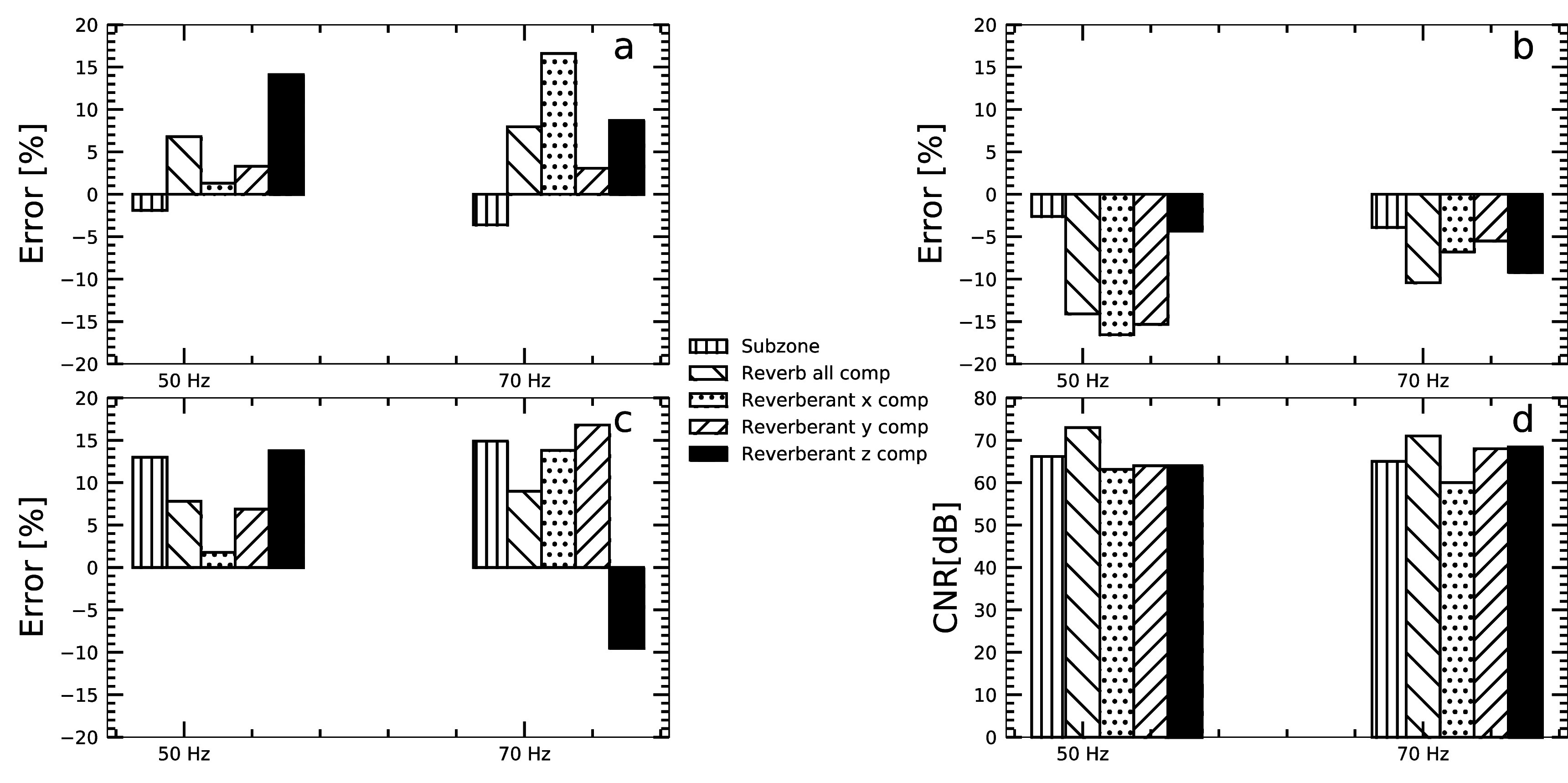
(a)–(c) shows the error incurred in shear modulus recovered from the 18 mm inclusion, 12 mm inclusion, and the background. Negative and positive error represents an underestimation and overestimation of the actual shear modulus, respectively. (d) shows the CNR of the recovered elastograms on a dB scale.

Figures 4(a), (b), (f), (g) shows reverberant and subzone elastograms corresponding to the displacement maps shown in figures 2(b)–(g). The 12- and 18 mm diameter inclusions were discernible in both sets of elastograms (reverberant (figure 4(b), (g)) and subzone (figure 4(a), (f))) when imaging at 50 and 70 Hz. Figures 4(c), (d), (e), (h), (i), (j) shows the reverberant elastograms computed by applying the reverberant method to individual components of displacements estimates when imaging at 50 Hz (figures 4(c), (d), (e)) and 70 Hz (figures 4(h), (i), (j)). Although both inclusions were visible in elastograms computed using individual reverberant displacement components, artifacts corrupted some elastograms, and the 12 mm diameter inclusion’s visibility depended on the displacement component employed. Figures 5(a), (b), (c) shows the accuracy of the recovered shear modulus of the inclusions (18 mm and 12 mm diameter) and surrounding background gel. The mean shear modulus of the 18 mm inclusion estimated from composite reverberant elastograms (created by averaging elastograms computed from individual displacements) and subzone elastograms was 6.8% higher and 1.9% lower respectively than its actual value when imaging at 50 Hz (figure 5(a)). For reverberant elastograms, the mean shear modulus of the 18 mm inclusion computed from the X, Y and Z components of displacements was 1.3%, 3.3% and 14.1% higher than the actual modulus. The mean shear modulus of the 12 mm diameter inclusion (figure 5(b)) estimated from subzone elastograms was 2.6% lower than the actual value when imaging at 50 Hz. Composite reverberant elastograms and elastograms computed from the X, Y and Z displacement components underestimated the shear modulus of the 12 mm diameter inclusion by 14.1%, 16.6%, 15.3 and 4.3%, respectively. We observed a similar trend for the estimated shear modulus of the background gel figure 5(c). In this case, the composite reverberant inversion was more accurate than the subzone inversion, and the most accurate reverberant elastograms were estimated from the Y displacement component. Figure 5(d) shows the CNR computed from the elastograms shown in figures 4(a)–(j). The CNR of subzone elastograms was comparable to those produced with the reverberant inversion method. The CNR of composite reverberant elastograms and those computed from the X displacement yielded the highest and lowest CNR, respectively, when imaging at 50 or 70 Hz.

### 3.2. Clinical studies

#### 3.2.1. Quantitative assessment displacement fields induced within the brain when imaging at 50 *Hz* and 70 *Hz*

**Figure 6.**
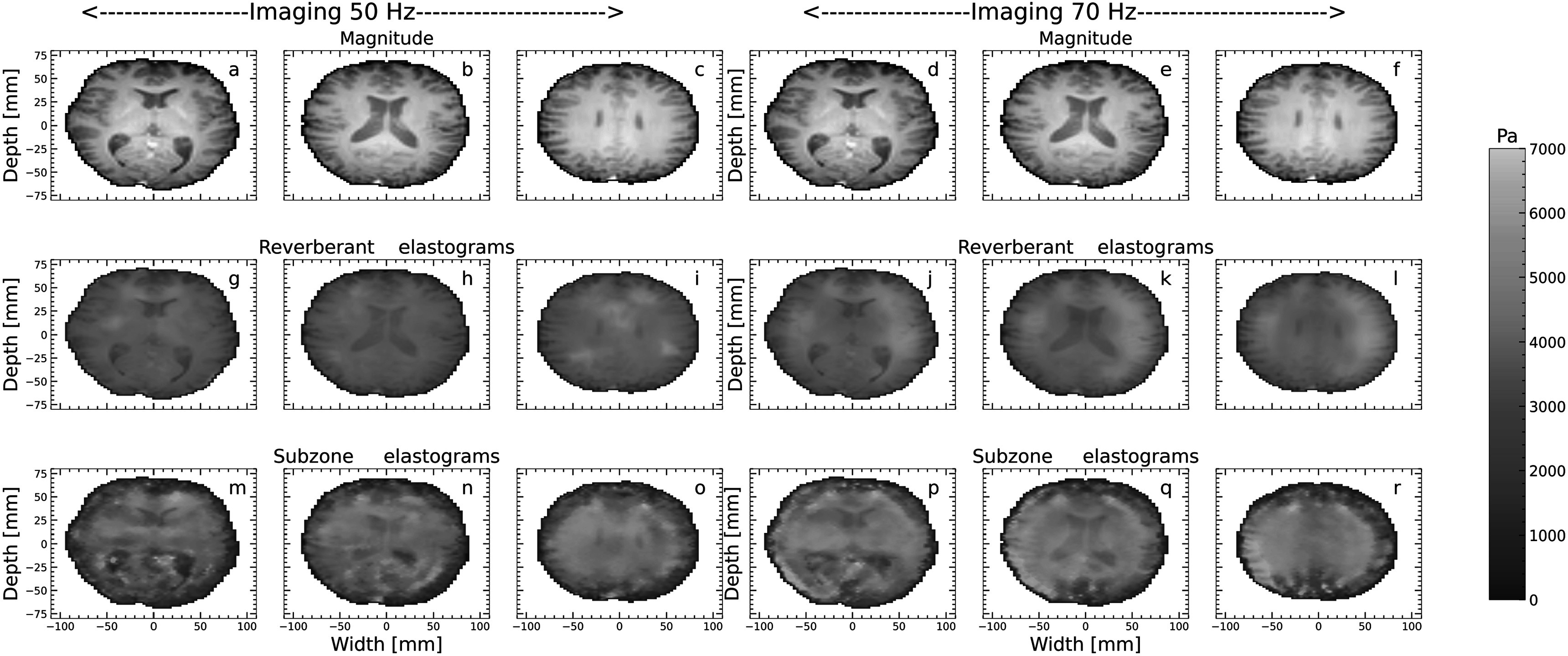
MR magnitude images (a)–(f) and shear modulus (g)–(r) elastograms recovered from a healthy volunteer when imaging at 50 Hz and 70 Hz. Recovered composite reverberant (g)–(i) and subzone (m)–(o) elastograms when imaging at 50 Hz. Composite reverberant (j)–(l) and subzone (p)–(r) elastograms recovered when imaging a 70 Hz.

Figures 6(a)–(r) shows representative examples of MR magnitude images and MR elastograms obtained from a healthy brain. Like the phantom studies, there was no statistically significant difference (*p* > 0.05) in wave fields obtained when imaging at 50 and 70 Hz. The levels of reverberance incurred in the brain were comparable to those incurred in the phantoms. More specifically, the percentage of reverberant pixels in each displacement component (X, Y, and Z) was 90%, 88% and 92% when imaging at 50 Hz and 91%, 87% and 93% when imaging at 70 Hz. The values were similar (88%–94%) for the other two brain data.

#### 3.2.2. Assessment of shear modulus values of different brain tissues from reverberant and subzone elastograms

Representative reverberant (figures 6(g)–(l)) and subzone figures 6(m)–(r) nt coronal positions within the brain when elastographic imaging was performed at 50 Hz (figures 6(g), (h), (i), (m), (n), (o)) and 70 Hz (figures 6(j), (k), (l), (p), (q), (r)). The global brain stiffnesses (GBT) estimated from subzone and reverberant elastograms shown in figure 6 were 2.5 ± 0.96 kPa and 2.3 ± 0.89 kPa, respectively, when imaging at 50 Hz. When imaging at 70 Hz, the GBT estimated from subzone and reverberant elastograms were 2.5 ± 0.82 kPa and 2.89 ±. 83 kPa, respectively.

**Figure 7.**
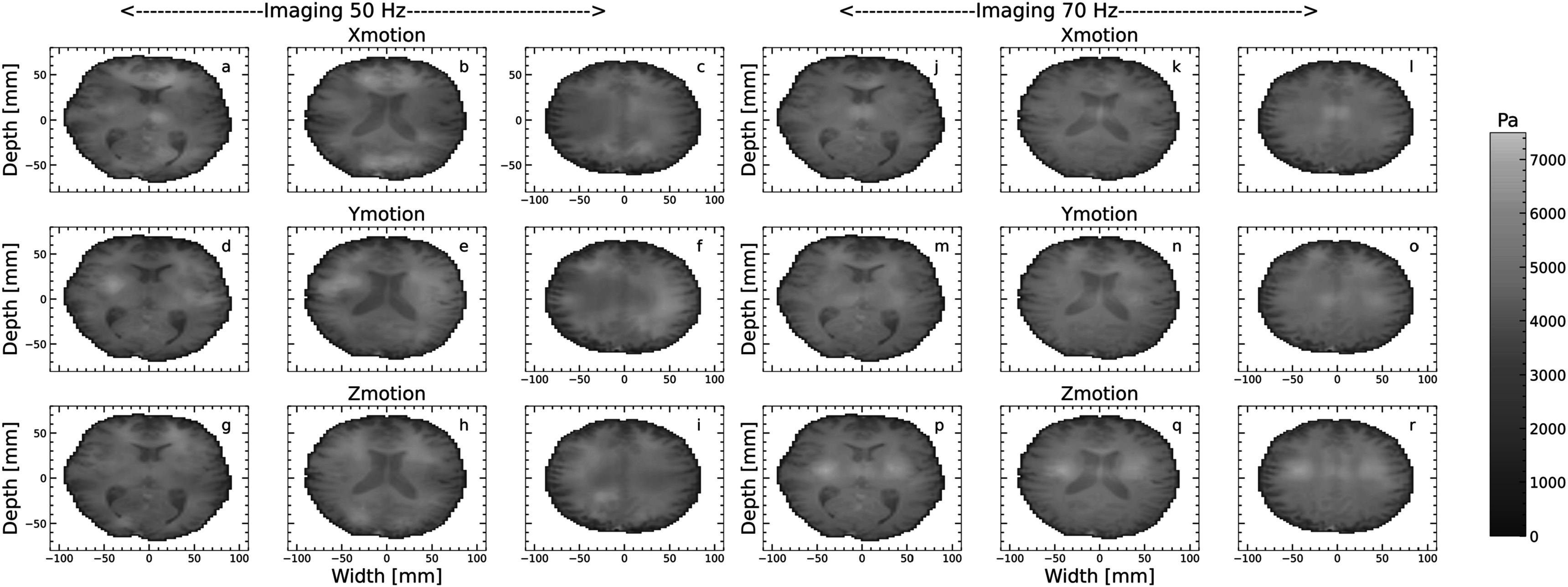
Reverberant elastograms recovered from individual displacement components when imaging the brain of a healthy volunteer at 50 Hz (a)–(i) and 70 Hz (j)–(r) displayed in units of [Pa]. Elastograms were computed from the X (a)–(c), Y (d)–(f), and Z(g)–(i) displacement components when imaging at 50 Hz at three different locations of the brain. The corresponding reverberant elastograms recovered from the X (j)–(l), Y (m)–(o), and Z(p)–(r) displacement components are also shown when imaging at 70 Hz.

**Figure 8.**
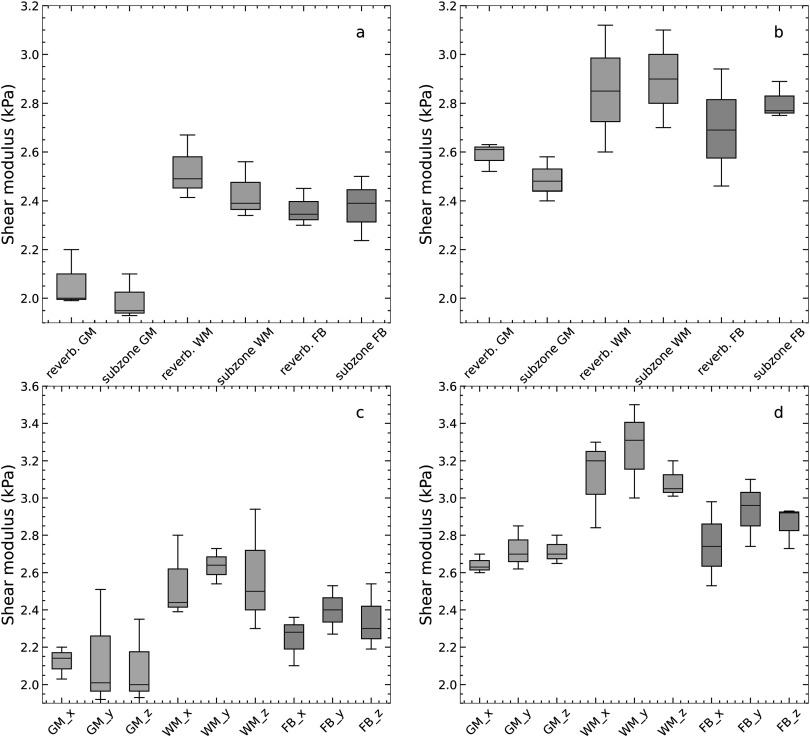
Box plots of the mean shear modulus of the whole brain (FB), gray matter (GM), and white matter (WM) for all subjects in this study. Showing the mean shear modulus computed by applying either the subzone inversion or the reverberant method (composite) to measured displacements when imaging at 50 Hz (a) and 70 Hz (b). Also shown are box plots of the mean shear modulus computed when the reverberant method was applied to individual displacement components denoted by the subscript of the displacement component employed when imaging at 50 Hz (c) and 70 Hz (d).

Figure 7 shows representative elastograms obtained from three different slices when the reverberant method was applied to individual displacement components when imaging at 50 Hz (figures 7(a)–(i)) and 70 Hz (figures 7(j)–(r)). The GBT estimated from X, Y, and Z motion component elastograms were 2.1 ± 0 .88 kPa, 2.4 ± 0.98 kPa, and 2.3 ± 0.99 kPa, respectively, when imaging at 50 Hz. When imaging at 70 Hz, the GBT estimated from the X, Y, and Z displacement components were 2.4 ± 0.82 kPa, 2.7 ± 0.75 kPa, and 2.89 ± 0.92 kPa, respectively. Figures 8(a), (b) shows box plots of the mean shear modulus of the whole brain, white matter, and gray matter estimated from composite reverberant and subzone elastograms for the volunteers employed in this study for 50 Hz and 70 Hz. At both frequencies, the global shear modulus of the brain was consistent with values reported in Murphy *et al (*Murphy *et al* 2013), Ingolf *et al (*Sack *et al* 2011), Matthew *et al (*Murphy *et al* 2011). and Arani *et al (*Arani *et al* 2015 *)*. The shear modulus of the white and gray matter was consistent with results previously reported (Zhang *et al* 2011, Johnson *et al* 2013a, 2013b). Figures 8(c), (d) shows box plots of the mean shear modulus of the whole brain, white matter, and gray matter estimated from reverberant elastograms computed from individual displacement components for 50 Hz and 70 Hz. Table 4 summarizes the shear modulus of the entire brain, white matter, and gray matter computed from reverberant and subzone elastograms for the volunteers employed in this study.

**Table 4.** Average Stiffness values for different brain regions using subzone and reverberant reconstruction approaches.

Volunteer								
Sex (M/F)	Age (years)	Frequency (Hz)	Method	Motion Component	% Reverberance	Gray matter (kPa)	White matter (kPa)	Whole brain (kPa)
F	73	50	Reverberant	X	89	2.03 ± 0.65	2.44 ± 0.45	2.36 ± 0.85
				Y	91	2.01 ± 0.57	2.54 ± 0.63	2.27 ± 0.82
				Z	92	1.93 ± 0.64	2.3 ± 0.59	2.19 ± 0.72
				all	N/A	2 ± 0.57	2.414 ± 0.54	2.345 ± 0.67
			subzone	all	N/A	1.93 ± 0.62	2.34 ± 0.68	2.237 ± 0.74
		70	Reverberant	X	90	2.6 ± 0.57	3.2 ± 0.63	2.74 ± 0.73
				Y	91	2.62 ± 0.42	3.5 ± 0.75	2.96 ± 0.91
				Z	89	2.70 ± 0.58	3.2 ± 0.57	2.92 ± 0.82
				all	N/A	2.63 ± 0.54	2.85 ± 0.69	2.69 ± 0.72
			subzone	all	N/A	2.58 ± 0.59	3.1 ± 0.69	2.75 ± 0.82
F	20	50	Reverberant	X	92	2.2 ± 0.65	2.8 ± 0.74	2.28 ± 0.79
				Y	90	1.92 ± 0.68	2.73 ± 0.64	2.53 ± 0.84
				Z	91	2 ± 0.53	2.94 ± 0.54	2.54 ± 0.73
				all	N/A	1.99 ± 0.58	2.67 ± 0.59	2.45 ± 0.78
			subzone	all	N/A	2.1 ± 0.66	2.56 ± 0.55	2.39 ± 0.69
		70	Reverberant	X	89	2.7 ± 0.69	3.3 ± 0.68	2.98 ± 0.79
				Y	93	2.7 ± 0.54	3.31 ± 0.58	3.10 ± 0.83
				Z	94	2.65 ± 0.43	3.01 ± 0.63	2.93 ± 0.95
				all	N/A	2.61 ± 0.54	3.12 ± 0.62	2.94 ± 0.75
			subzone	all	N/A	2.48 ± 0.64	2.90 ± 0.53	2.77 ± 0.81
M	22	50	Reverberant	X	90	2.14 ± 0.79	2.39 ± 0.93	2.1 ± 0.88
				Y	88	2.51 ± 1.07	2.67 ± 1.1	2.4 ± 0.98
				Z	92	2.35 ± 1.05	2.50 ± 0.90	2.3 ± 0.99
				all	N/A	2.2 ± 0.88	2.49 ± 0.88	2.3 ± 0.89
			subzone	all	N/A	1.95 ± 0.94	2.39 ± 0.93	2.5 ± 0.96
		70	Reverberant	X	91	2.63 ± 0.71	2.85 ± 0.68	2.49 ± 0.82
				Y	87	2.85 ± 0.82	3 ± 0.74	2.74 ± 0.75
				Z	93	2.80 ± 0.81	3.05 ± 0.84	2.89 ± 0.92
				all	N/A	2.4 ± 0.82	2.7 ± 0.75	2.89 ± 0.83
			subzone	all	N/A	2.52 ± 0.79	2.6 ± 0.86	2.5 ± 0.82

## 4. Discussion

This study introduced an analog of the reverberant shear wave elastographic imaging technique previously developed for ultrasound elastography (Parker *et al* 2017), now adapted for MRE. Reverberant elastography assumes a superposition of waves generated by multiple point sources, and reflections will generate a complex 3D shear wave field. Under these conditions, the two-dimensional autocorrelation function of the complex wave field and the phase gradient provides fast and accurate estimates of the wavenumber from which shear wave speed and modulus are derived. Reverberant elastography has been demonstrated with ultrasound (Parker *et al* 2017) and optical coherence tomography (Zvietcovich *et al* 2019, Ge *et al* 2022) using multiple point drivers. However, to our knowledge, this is the first reported study demonstrating its utility with MRE and its feasibility in the brain using a single mechanical driver. We used the goodness of fit performance metric to quantify the degree of reverberance in different displacement components. We also studied three healthy volunteers to assess how reverberant elastography performs within the clinical setting. The primary findings of this investigation were as follows:

1. A single mechanical source generates a complex wave field (reverberant) in constrained phantoms, quantified by assessing how well the measured autocorrelation function matched the theoretically derived one. Applying the reverberant shear modulus estimation method to the resulting shear wave fields produced elastography with variable accuracy (83.2%–98.7%) and CNR ranging from 63.1 to 73 dB (figure 5).
2. The performance (accuracy and CNR) of subzone and composite reverberant elastograms were comparable. However, the performance of reverberant elastograms degraded marginally when reverberant elastograms were computed from a single displacement component (figure 5).
3. The reverberant reconstruction method produced meaningful elastograms when applied to one displacement component, but performance varied based on the displacement component used. We observed the best performance when reconstructions were performed using all displacement components. This raises concerns about the usefulness of reverberant elastograms produced from a single displacement component obtained from a partially reverberant field.
4. The mean shear modulus of the whole brain, white, and *gray* matter estimated from composite reverberant and subzone elastograms obtained from healthy volunteers were consistent with previously reported shear modulus estimates of the healthy brain.

In ultrasound-reverberant elastography, elastograms acquired at higher frequencies were superior to those obtained at lower frequencies (Parker *et al* 2017, Ormachea *et al* 2019); in this study, the quality of elastograms produced at 50 and 70 Hz was similar. Figure 2 demonstrates the impact of attenuation on the displacements when images are acquired at 50 Hz and 70 Hz. Despite the reduction in shear wave amplitude, the displacement amplitudes at 70 Hz were high enough to produce reasonable elastograms. Although we increased the amplitude of shear waves when imaging at 70 Hz, however, since the mechanical driver is pneumatic, the amplitude of the shear wave at the passive driver is likely to be much less than at the active driver. Therefore, future studies will measure the amplitude shear waves at both the active and passive drivers during imaging.

The composite elastograms and those computed from the individual displacement components overestimated the modulus of the 18 mm diameter inclusion (see figure 5(a)). The 12 mm diameter inclusion shear modulus was underestimated in all cases (see figure 5(b)). The cause of this behavior is unclear, but it may be due to using a suboptimal kernel or a partially reverberant field. We expect that using larger kernels will increase the CNR but cause the modulus of smaller inclusion to be underestimated. Employing a larger kernel would also reduce the background noise observed in the elastograms (see figure 5(c)). In contrast, smaller kernels should provide more accurate results, although with a lower CNR. Further work is needed to fully understand the kernel size’s impact on the experiment’s performance and to determine the underlying cause of the observed behavior.

Model-based inversion approaches need all three displacement components to compute shear modulus precisely (Doyley 2012). Researchers have demonstrated theoretically that computing shear modulus with fewer components reduces the accuracy of the resulting elastograms (Skovoroda *et al* 1994). Sampling error and poor ultrasound penetration, especially in patients with high body mass index (Zhao *et al* 2014) cause the diagnostic performance of ultrasound elastography for detecting and staging liver fibrosis to be lower than that achieved with MRE (Li *et al* 2021). Figure 5 demonstrates that reverberant shear wave elastography can create reasonably accurate elastograms (on the order of 16.8% error) from a single displacement component. Although shear elastograms computed from individual displacement components may differ in appearance (see figure 7), the average modulus computed from each brain region was similar (see figure 8). Using only a single displacement component when performing reverberant MR elastography is not recommended due to inconsistent results obtained with different displacement components. We recommend that composite reverberant elastograms be computed from all three displacement components to obtain the best results.

Researchers have demonstrated that longitudinal waves make it difficult to reconstruct shear modulus from a single displacement field (Honarvar *et al* 2013). The presence of longitudinal waves could be one reason the performance of reverberant elastograms varied with different components of displacements. The curl operator is typically used to minimize the impact of longitudinal waves. The bandpass filter employed in this study was equivalent to computing the vector curl of the complex wave field (Sinkus *et al* 2005). Studies performed on a constrained gelatin phantom and the brains of healthy volunteers demonstrated that reverberant shear wave fields could be generated using standard elastographic imaging equipment (i.e. a single mechanical driver). Besides mechanical sources, other factors, such as the position of the external reflectors, size of the mechanical sources, shear wave attenuation, vibration amplitude, etc, will dictate the degree of reverberance induced within tissues. The impact of these variables on reverberant fields induced in soft tissue is beyond the scope of this work.

Nevertheless, intuitively, we expect to use fewer mechanical sources to generate a fully reverberant shear wave field in constrained organs such as the brain compared to unconstrained organs. Using multiple shakers can direct waves in different directions. Furthermore, the vibration from these sources can be out of phase to reduce the formation of standing waves. Therefore, we plan to investigate the clinical performance of reverberant brain elastograms obtained using a mechanical actuation system like that described in (Anderson *et al* 2016, Li *et al* 2021), which employs multiple mechanical sources.

A limitation of this study is that we did not perform any imaging on an unconstrained phantom or organ. To address this, we plan to compare the performance of the shear modulus estimation method on both constrained and unconstrained phantoms. Although figures 6 and 7 demonstrated that the reverberant elastography method produces clinically useful elastograms, the spatial resolution of acquisition may be another factor that may affect the shear modulus. In this study, we used a 2.5 mm isotropic voxel size. Curtis *et al (*Johnson *et al* 2014 *)*. used 2 mm, and (Zhang *et al* 2011 *)*. used 3 mm in their studies. Reconstructions from only one component of motion displayed consistent shear modulus values (see table 4). We are currently conducting studies on a larger cohort of patients to evaluate the clinical performance of the reverberant elastograms; the results we will report in a future publication. Although the reconstructions from reverberant elastography have a lower spatial resolution, the reconstruction times are much faster. The shorter reconstruction times and accurate results make the technique well-suited for clinical studies in the brain.

## 5. Conclusion

This study corroborated the hypothesis that reverberant elastography produces reliable shear modulus elastograms of constrained organs, such as the brain, with a single mechanical driver. Studies performed on a brain-shape phantom demonstrated that the accuracy of reverberant elastograms computed using all three displacement components was comparable to those calculated using the subzone inversion method. The clinical study results were consistent with those of the phantom study (i.e. the performance of reverberant and subzone elastograms was comparable) and sufficiently encouraging enough to warrant further evaluation with a larger cohort of subjects.

## Data Availability

The data that support the findings of this study is available at https://doi.org/10.5281/zenodo.7643845.

## Ethical statement

The University of Delaware Institutional Review Board approved the imaging protocol used in this study. IRB approval number 1436845. All human studies were conducted in accordance with the principles embodied in the Declaration of Helsinki and in accordance with local statutory requirements.

## References

[pmbacbbb7bib1] Anderson A T (2016). Observation of direction-dependent mechanical properties in the human brain with multi-excitation MR elastography. J. Mech. Behav. Biomed. Mater..

[pmbacbbb7bib2] Arani A (2015). Measuring the effects of aging and sex on regional brain stiffness with MR elastography in healthy older adults. Neuroimage..

[pmbacbbb7bib3] Barnhill E (2018). Heterogeneous Multifrequency Direct Inversion (HMDI) for magnetic resonance elastography with application to a clinical brain exam. Med. Image Anal..

[pmbacbbb7bib4] Brock M (2015). Impact of real-time elastography on magnetic resonance imaging/ultrasound fusion guided biopsy in patients with prior negative prostate biopsies. J. Urol..

[pmbacbbb7bib5] Chaze C A (2019). Altered brain tissue viscoelasticity in pediatric cerebral palsy measured by magnetic resonance elastography. Neuroimage Clin..

[pmbacbbb7bib6] Clayton E H, Genin G M, Bayly P V (2012). Transmission, attenuation and reflection of shear waves in the human brain. J. R. Soc. Interface.

[pmbacbbb7bib7] Doyley M M (2003). Thresholds for detecting and characterizing focal lesions using steady-state MR elastography. Med. Phys..

[pmbacbbb7bib8] Doyley M M (2010). The performance of steady-state harmonic magnetic resonance elastography when applied to viscoelastic materials. Med. Phys..

[pmbacbbb7bib9] Doyley M M (2012). Model-based elastography: a survey of approaches to the inverse elasticity problem. Phys. Med. Biol..

[pmbacbbb7bib10] Doyley M M, Meaney P M, Bamber J C (2000). Evaluation of an iterative reconstruction method for quantitative elastography. Phys. Med. Biol..

[pmbacbbb7bib11] Dresner M A (2001). Magnetic resonance elastography of skeletal muscle. J. Magn. Reson. Imaging.

[pmbacbbb7bib12] Freimann F B (2012). Alteration of brain viscoelasticity after shunt treatment in normal pressure hydrocephalus. Neuroradiology..

[pmbacbbb7bib13] Fung Y C (1981). Biomechanics: Mechanical Properties of Living Tissue.

[pmbacbbb7bib14] Ge G R (2022). Assessing corneal cross-linking with reverberant 3D optical coherence elastography. J. Biomed. Opt..

[pmbacbbb7bib15] Hiscox L V (2016). Magnetic resonance elastography (MRE) of the human brain: technique, findings and clinical applications. Phys. Med. Biol..

[pmbacbbb7bib16] Honarvar M (2013). Curl-based finite element reconstruction of the shear modulus without assuming local homogeneity: time harmonic case. IEEE Trans. Med. Imaging.

[pmbacbbb7bib17] Hu L (2020). Requirements for accurate estimation of shear modulus by magnetic resonance elastography: a computational comparative study. Comput. Methods Programs Biomed..

[pmbacbbb7bib18] Hu L, Shan X (2020). Enhanced complex local frequency elastography method for tumor viscoelastic shear modulus reconstruction. Comput. Methods Programs Biomed..

[pmbacbbb7bib19] Johnson C L (2013a). Local mechanical properties of white matter structures in the human brain. Neuroimage..

[pmbacbbb7bib20] Johnson C L (2013b). Magnetic resonance elastography of the brain using multishot spiral readouts with self-navigated motion correction. Magn. Reson. Med..

[pmbacbbb7bib21] Johnson C L (2014). 3D multislab, multishot acquisition for fast, whole-brain MR elastography with high signal-to-noise efficiency. Magn. Reson. Med..

[pmbacbbb7bib22] Knutsson H, Westin C-F, Granlund G (1994). Local multiscale frequency and bandwidth estimation, in. Image Processing, 1994. Proc. ICIP-94., IEEE Int. Conf..

[pmbacbbb7bib23] Kruse S A (2000). Tissue characterization using magnetic resonance elastography: preliminary results [In Process Citation]. Phys. Med. Biol..

[pmbacbbb7bib24] Kruse S A (2008). Magnetic resonance elastography of the brain. Neuroimage..

[pmbacbbb7bib25] Li B N (2014). Evaluation of robust wave image processing methods for magnetic resonance elastography. Comput. Biol. Med..

[pmbacbbb7bib26] Li M S (2021). Tomoelastography based on multifrequency MR elastography for prostate cancer detection: comparison with multiparametric MRI. Radiology.

[pmbacbbb7bib27] Lipp A (2013). Cerebral magnetic resonance elastography in supranuclear palsy and idiopathic Parkinson’s disease. Neuroimage Clin..

[pmbacbbb7bib28] Manduca A (1996). Local wavelength estimation for magnetic-resonance elastography. Proc 3rd IEEE Int. Conf. Image Process..

[pmbacbbb7bib29] Manduca A (2001). Magnetic resonance elastography: non-invasive mapping of tissue elasticity. Med. Imaging Anal..

[pmbacbbb7bib30] Mariappan Y K (2014). Estimation of the absolute shear stiffness of human lung parenchyma using (1) H spin echo, echo planar MR elastography. J. Magn. Reson. Imaging.

[pmbacbbb7bib31] McGarry M D (2011). An octahedral shear strain-based measure of SNR for 3D MR elastography. Phys. Med. Biol..

[pmbacbbb7bib32] McKnight A L (2002). MR elastography of breast cancer: preliminary results. AJR Am J Roentgenol..

[pmbacbbb7bib33] Murphy M C (2011). Decreased brain stiffness in Alzheimer’s disease determined by magnetic resonance elastography. J. Magn. Reson. Imaging.

[pmbacbbb7bib34] Murphy M C (2013). Measuring the characteristic topography of brain stiffness with magnetic resonance elastography. PLoS One.

[pmbacbbb7bib35] Muthupillai R (1995). Magnetic-resonance elastography by direct visualization of propagating acoustic strain waves. Science.

[pmbacbbb7bib36] Oliphant T E (2001). Complex-valued stiffness reconstruction for magnetic resonance elastography by algebraic inversion of the differential equation. Magn. Reson. Med..

[pmbacbbb7bib37] Ormachea J, Castaneda B, Parker K J (2018). Shear wave speed estimation using reverberant shear wave fields: implementation and feasibility studies. Ultrasound Med. Biol..

[pmbacbbb7bib38] Ormachea J, Parker K J (2021). Reverberant shear wave phase gradients for elastography. Phys. Med. Biol..

[pmbacbbb7bib39] Ormachea J, Parker K J, Barr R G (2019). An initial study of complete 2D shear wave dispersion images using a reverberant shear wave field. Phys. Med. Biol..

[pmbacbbb7bib40] Papazoglou S (2008). Algebraic Helmholtz inversion in planar magnetic resonance elastography. Phys. Med. Biol..

[pmbacbbb7bib41] Parker K J (2017). Reverberant shear wave fields and estimation of tissue properties. Phys. Med. Biol..

[pmbacbbb7bib42] Parker K J, Doyley M M, Rubens D J (2011). Imaging the elastic properties of tissue: the 20 year perspective. Phys. Med. Biol..

[pmbacbbb7bib43] Parker K W, Doyely M M, Ruben D (2010). Elastography. Phys. Med. Biol..

[pmbacbbb7bib44] Patel B K (2021). MR elastography of the breast: evolution of technique, case examples, and future directions. Clin Breast Cancer..

[pmbacbbb7bib45] Sack I (2011). The influence of physiological aging and atrophy on brain viscoelastic properties in humans. PLoS One.

[pmbacbbb7bib46] Sack I (2013). Structure-sensitive elastography: on the viscoelastic powerlaw behavior of *in vivo* human tissue in health and disease. Soft Matter..

[pmbacbbb7bib47] Scott J M (2020). Artificial neural networks for magnetic resonance elastography stiffness estimation in inhomogeneous materials. Med. Image Anal..

[pmbacbbb7bib48] Sinkus R (2000). High-resolution tensor MR elastography for breast tumour detection. Phys. Med. Biol..

[pmbacbbb7bib49] Sinkus R (2005). Viscoelastic shear properties of *in vivo* breast lesions measured by MR elastography. Magn. Reson. Imaging.

[pmbacbbb7bib50] Sinkus R (2007). MR elastography of breast lesions: understanding the solid/liquid duality can improve the specificity of contrast-enhanced MR mammography. Magn. Reson. Med..

[pmbacbbb7bib51] Skovoroda A R (1994). Theoretical-analysis and verification of ultrasound displacement and strain imaging. IEEE Trans. Ultrason. Ferroelectr. Freq. Control.

[pmbacbbb7bib52] Smith D R (2020). Multi-excitation magnetic resonance elastography of the brain: wave propagation in anisotropic white matter. J. Biomech. Eng..

[pmbacbbb7bib53] Smith S M (2004). Advances in functional and structural MR image analysis and implementation as FSL. Neuroimage..

[pmbacbbb7bib54] Strang G (2016). Introduction to Linear Algebra.

[pmbacbbb7bib55] Streitberger K J (2011). *In vivo* viscoelastic properties of the brain in normal pressure hydrocephalus. NMR Biomed..

[pmbacbbb7bib56] Techavipoo U, Varghese T (2005). Improvements in elastographic contrast-to-noise ratio using spatial-angular compounding. Ultrasound Med. Biol..

[pmbacbbb7bib57] Van Houten E E (1999). An overlapping subzone technique for MR-based elastic property reconstruction. Magn. Reson. Med..

[pmbacbbb7bib58] Van Houten E E (2001a). Three-dimensional subzone-based reconstruction algorithm for MR elastography. Magn. Reson. Med..

[pmbacbbb7bib59] Van Houten E E W (2001b). Three-dimensional subzone-based reconstruction algorithm for MR elastography. Magn. Reson. Med..

[pmbacbbb7bib60] Venkatesh S K, Yin M, Ehman R L (2013). Magnetic resonance elastography of liver: technique, analysis, and clinical applications. J. Magn. Reson. Imaging.

[pmbacbbb7bib61] Weaver J B (2001). Magnetic resonance elastography using 3D gradient echo measurements of steady-state motion. Med. Phys..

[pmbacbbb7bib62] Wuerfel J (2010). MR-elastography reveals degradation of tissue integrity in multiple sclerosis. Neuroimage..

[pmbacbbb7bib63] Yin M (2007). Assessment of hepatic fibrosis with magnetic resonance elastography. Clin. Gastroenterol. Hepatol..

[pmbacbbb7bib64] Zhang J (2011). Viscoelastic properties of human cerebellum using magnetic resonance elastography. J. Biomech..

[pmbacbbb7bib65] Zhao H (2014). Noninvasive assessment of liver fibrosis using ultrasound-based shear wave measurement and comparison to magnetic resonance elastography. J. Ultrasound Med..

[pmbacbbb7bib66] Zhao Z (2018). Robust 2D phase unwrapping algorithm based on the transport of intensity equation. Meas. Sci. Technol..

[pmbacbbb7bib67] Zvietcovich F (2019). Reverberant 3D optical coherence elastography maps the elasticity of individual corneal layers. Nat. Commun..

